# Supplementary figures and images for: Topography and environmental deficiencies are associated with chikungunya virus exposure in urban informal settlements in Salvador, Brazil
Source: PLoS Negl Trop Dis. 2025 Sep 5;19(9):e0013477. doi: 10.1371/journal.pntd.0013477 (PMC12440227; doi:10.1371/journal.pntd.0013477)

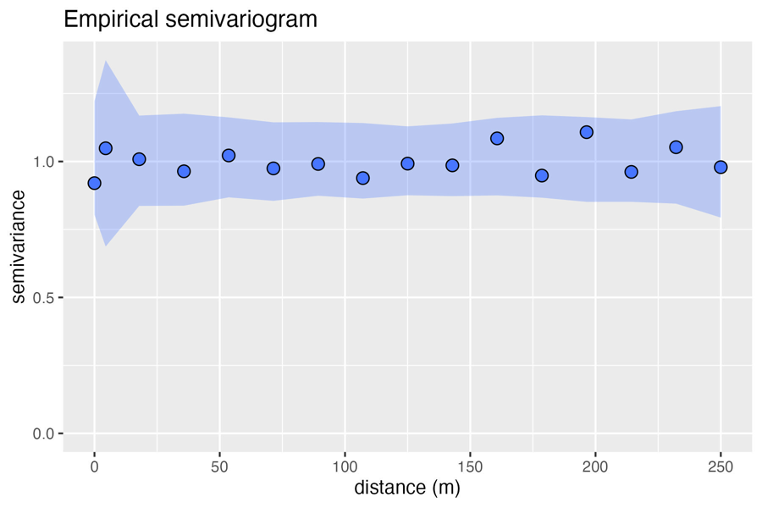

Supplement: S3 Fig — (TIFF) [file pntd.0013477.s003.tiff]
