# Supplementary material for: Topography and environmental deficiencies are associated with chikungunya virus exposure in urban informal settlements in Salvador, Brazil
Source: PLoS Negl Trop Dis. 2025 Sep 5;19(9):e0013477. doi: 10.1371/journal.pntd.0013477 (PMC12440227; doi:10.1371/journal.pntd.0013477)
Supplement: Attachment — Submitted filename: CHIKV Paper Comments (1).docx [file pntd.0013477.s012.docx]

# OVERALL STATEMENT

The manuscript by Catherine Travis and co-authors examines Chikungunya virus (CHIKV) exposure risks in four urban informal settlements within a large Brazilian city. It utilizes a cross- sectional serological survey and environmental data mapping to identify the contributions of individual, household, and environmental factors for seropositivity. The authors correlate the seroprevalence data with environmental factors. The authors reported that they found 9.2% testing positive for CHIKV-specific antibodies and highlighted the exposure risk in low-income settlements. The theoretical aspects almost substantiate the investigation performed by the authors. Therefore, I would recommend for publication with major revisions.

# COMMENTS TO AUTHORS

**MAJOR ESSENTIALS REVISION**

1. In the abstract the serology data were collected from 1217 participants and in the results 1318 participants were mentioned. Kindly clarify this number.
2. Did the authors check for IgM also since IgM is the first antibody that is formed during a viral infection. IgG levels are elevated only during secondary infection. What was the logic behind testing for IgG instead of IgM for serological purposes? Testing for IgG alone will not tell us the exact exposure to CHIKV. The exact exposure will be calculated with IgG and IgM titers.
3. The authors can add the sensitivity of the IgG Kit used in the seroprevalence study.
4. Were the samples tested for IgG in duplicates or triplicates before the final results.
5. The authors collected Environmental data in 2019, but the serological data were from 2018, which could introduce discrepancies in the analysis.

# MINOR ESSENTIAL REVISIONS

1. Introduction> Second paragraph

A sentence cannot be started with “Because”. Kindly correct all sentences like this in the manuscript. - “Because of concurrent outbreaks of CHIKV, dengue (DENV), and Zika viruses (ZIKV), health surveillance systems in Brazil are frequently overwhelmed and there is difficulty measuring the true burden of CHIKV”

2. Introduction > Fifth paragraph

This sentence is grammatically incorrect and should be modified accordingly. “Capturing fine-scale variation in environmental features is consequently important for estimating the effect of environmental	”

3. Methods > Second paragraph

This sentence is grammatically incorrect and should be modified accordingly. “First, a full census of the study areas was conducted and all individuals ≥5 years of age who slept	”

4. Methods > Serological Evaluation

“Held at” should be replaced by appropriate word in the sentence “After aliquoting serum from centrifuged samples, the serums were held at −20°C and tested using a

commercial	”

With warm regards

Shama Parveen
