# Supplementary material for: Topography and environmental deficiencies are associated with chikungunya virus exposure in urban informal settlements in Salvador, Brazil
Source: PLoS Negl Trop Dis. 2025 Sep 5;19(9):e0013477. doi: 10.1371/journal.pntd.0013477 (PMC12440227; doi:10.1371/journal.pntd.0013477)
Supplement: Attachment — Submitted filename: Response to Reviewers.docx [file pntd.0013477.s014.docx]

Max Eyre

**Assistant Professor**

max.eyre@lshtm.ac.uk

­­——

London School of Hygiene & Tropical Medicine

Keppel Street, London WC1E 7HT

4^th^ June 2025

Dear Editors,

Please find below our responses to reviewer comments.

Many thanks,

Yours faithfully,

 

Max Eyre

**MAJOR ESSENTIAL REVISIONS**

**Reviewer Comment 1:**

**In the abstract the serology data were collected from 1217 participants and in the results 1318 participants were mentioned. Kindly clarify this number.**

Response 1:

Thank you for highlighting this inconsistency. The number 1217 initially listed in the abstract was not correct and seems to be a result of incorrect finger placement when typing. The correct number of participants is 1318, and this has been reflected in line 12.

**Reviewer Comment 2:**

**Did the authors check for IgM also since IgM is the first antibody that is formed during a viral infection. IgG levels are elevated only during secondary infection. What was the logic behind testing for IgG instead of IgM for serological purposes? Testing for IgG alone will not tell us the exact exposure to CHIKV. The exact exposure will be calculated with IgG and IgM titers.**

Response 2:

Thank you for raising this point. In the case of CHIKV, the statement that IgG levels are elevated only during secondary infection is not correct. [Silva and Dermody (2017)](https://pmc.ncbi.nlm.nih.gov/articles/PMC5330729/) wrote about IgG and IgM of CHIKV in humans and stated “In humans, IgM levels are detected within 5–7 days after the onset of symptoms ([153](https://pmc.ncbi.nlm.nih.gov/articles/PMC5330729/#B153)), peak several weeks after infection ([154](https://pmc.ncbi.nlm.nih.gov/articles/PMC5330729/#B154)), and begin to wane over the next several months ([154](https://pmc.ncbi.nlm.nih.gov/articles/PMC5330729/#B154)). An IgG response can be detected approximately 7–10 days after onset of illness, often after viremia has been cleared ([37](https://pmc.ncbi.nlm.nih.gov/articles/PMC5330729/#B37), [154](https://pmc.ncbi.nlm.nih.gov/articles/PMC5330729/#B154)–[156](https://pmc.ncbi.nlm.nih.gov/articles/PMC5330729/#B156)).” While testing for IgM can allow for differentiation between more and less recent exposures than just using IgG (which captures all previous exposures), this makes relatively little difference for our analysis as the data were collected in 2018 and CHIKV transmission only commenced in Salvador in 2015. Consequently, our IgG approach allows us to capture all infections that took place 2015-2018, allowing us to characterize the drivers of transmission during this period. Given there was no significant evidence of a large CHIKV outbreak in Salvador until a relatively short period before data collection we believe that using IgM would not have resulted in very different results anyway. I have added clarification of this point in lines 117-119: “There was no evidence of significant CHIKV transmission in Salvador before 2015 and these IgG results are consequently representative of recent cases occurring in the period 2015-2018”.

**Reviewer Comment 3:**

**The authors can add the sensitivity of the IgG Kit used in the seroprevalence study.**

Response 3:

Thanks for pointing this out, we have added additional information regarding the sensitivity and specificity of the test kit used in line 117 “anti-CHIKV IgG ELISA with >90% Relative Sensitivity and Relative Specificity [15].”

**Reviewer Comment 4:**

**Were the samples tested for IgG in duplicates or triplicates before the final results.**

Response 4:

Thanks for this question, if the first test found an indeterminant result, the sample was tested once more. We have specified this information in lines 122-123 “Samples that first returned indeterminant results were retested and the second result was considered final [4].”

**Reviewer Comment 5:**

**The authors collected Environmental data in 2019, but the serological data were from 2018, which could introduce discrepancies in the analysis.**

Response 5:

Thank you for raising this point. These remotely sensed variables e.g. land cover, and elevation are minimally variable over the period of around 5 years. In this study our exposure window is 3 years in duration (2015-2018). Clarification of this has been added to lines 141-143: “. While the satellite images were taken one year after the data collection period, these environmental data are minimally variable over and are still representative of the exposure window.”

**MINOR ESSENTIAL REVISIONS**

**Reviewer Comment 6:**

**Introduction> Second paragraph**

**A sentence cannot be started with “Because”. Kindly correct all sentences like this in the manuscript. - “Because of concurrent outbreaks of CHIKV, dengue (DENV), and Zika viruses (ZIKV), health surveillance systems in Brazil are frequently overwhelmed and there is difficulty measuring the true burden of CHIKV”**

Response 6:

Thank you for raising this. In line 55, “Because of” has been corrected to “Due to”.

**Reviewer Comment 7:**

**Introduction > Fifth paragraph**

**This sentence is grammatically incorrect and should be modified accordingly. “Capturing fine-scale variation in environmental features is consequently important for estimating the effect of environmental	”**

Response 7:

Thank you for raising this, we have separated that sentence into two and corrected the grammar issues. From lines 76-78 the sentences now read “Consequently, capturing fine-scale variation in environmental features helps to estimate environmental effect alone and in conjunction with individual and household factors. Accurate estimation of the effect environmental features can provide critical data in targeting of vector control within communities.”

**Reviewer Comment 8:**

**Methods > Second paragraph**

**This sentence is grammatically incorrect and should be modified accordingly. “First, a full census of the study areas was conducted and all individuals ≥5 years of age who slept	”**

Response 8:

Thank you for flagging this issue. We have edited the sentence in lines 100-102 to “First, study canvassers conducted a full census of the study areas. All individuals ≥5 years of age who slept ≥3 nights per week within the study areas and had lived there for at least 6 months were invited to participate in the study.”

**Reviewer Comment 9:**

**Methods > Serological Evaluation**

**“Held at” should be replaced by appropriate word in the sentence “After aliquoting serum from centrifuged samples, the serums were held at −20°C and tested using a commercial	”**

Response 9:

Thank you for highlighting this, we have changed the wording in line 116 from “held at” to “stored at”.

**REVIEWERS 1&3:**

**Reviewers' Comments:**

Reviewer's Responses to Questions

**Key Review Criteria Required for Acceptance?**

As you describe the new analyses required for acceptance, please consider the following:

**Methods**

-Are the objectives of the study clearly articulated with a clear testable hypothesis stated?

-Is the study design appropriate to address the stated objectives?

-Is the population clearly described and appropriate for the hypothesis being tested?

-Is the sample size sufficient to ensure adequate power to address the hypothesis being tested?

-Were correct statistical analysis used to support conclusions?

-Are there concerns about ethical or regulatory requirements being met?

**Reviewer #1:**

**Reviewer Comment 10:**

**The article addresses a relevant topic regarding the connection between risk factors and chikungunya prevalence. However a few details could improve the readability and clarity.

The methodology section provides valuable details on the data sources used in the study.**
**In line 95, although an encyclopedia [12] can provide general definitions, I suggest substituting it with peer-reviewed scientific sources that empirically describe the socio-environmental conditions of favelas or informal settlements in Brazil, like reference [33]. This would enhance the study's reliability and alignment with the empirical rigor expected in public health research.**
Response 10:

Thank you for pointing this out, we have adjusted the reference supporting the information in lines 92-94 to the article cited

Lilford, R., Kyobutungi, C., Ndugwa, R., Sartori, J., Watson, S. I., Sliuzas, R., Kuffer, M., Hofer, T., Porto de Albuquerque, J., & Ezeh, A. (2019). Because space matters: conceptual framework to help distinguish slum from non-slum urban areas. BMJ global health, 4(2), e001267. https://doi.org/10.1136/bmjgh-2018-001267

**Reviewer Comment 11:**

**To improve clarity, kindly specify in Supplemental Table 1 the category to which each variable belongs.**
Response 11:

Thank you for raising this. In Supplemental Table 1, headings “Individual Variables”, “Household Variables”, and “Environmental Variables” have been added to improve clarity.

**Reviewer Comment 12:**

**In line 132, kindly indicate the version of QGIS and the Semi-Automatic Classification Plugin utilized in the analysis.**
Response 12: Thank you for pointing this out, in line 137 we added information of the version numbers. Lines 135 to 137 now read “Land cover rasters were created from fine-scale WorldView-3 satellite images taken in February 2019 (resolution of 0.3m by 0.3m) using the Semi-Automatic Classification Plugin 8.5.0 in QGIS LTR 3.16 [17].”

**Reviewer Comment 13;**

**Additionally, it would be helpful to clarify the rationale behind using a satellite image from a different period than the sampling timeframe. Could the authors provide justification for this choice and include it in the Methods section? Ensuring consistency between the imagery and field data collection period would strengthen the methodological coherence of the study.**

Response 13: Thank you for raising this. This was adjusted in Response 5.

**Reviewer #3:**

**Reviewer Comment 14:**

**The manuscript by Travis and colleagues aims to identify individual, household, and environmental factors that may contribute to exposure to Chikungunya virus (CHIKV) in four communities in Salvador, a large Brazilian city. To this end, a cross-sectional serological survey was conducted, and blood samples and data were collected from 1318 participants, as well as a measurement of the environmental characteristics of the communities, between March and October 2018.

The authors obtained approval from the ethics committee of the institution where the study was conducted and the National Commission for Research Ethics, Brazilian Ministry of Health; as well as the free and informed consent form of the participants.

My experience with statistical analysis is limited, so my observations and interpretations may not be as in-depth in this regard.**

Response 14:

Thank you for any and all input you have provided.

**Results**

-Does the analysis presented match the analysis plan?

-Are the results clearly and completely presented?

-Are the figures (Tables, Images) of sufficient quality for clarity?

**Reviewer #1:**

**Reviewer Comment 15:**

**15.1 In Table 1, the authors list the category "Boot access" with its subcategories (No, Own boots, Can borrow boots), whereas the other variables are displayed in a binary Yes/No format. Could you clarify the rationale for using subcategories in this specific case? To ensure consistency, I recommend standardizing all variables as dichotomous.

15.2 The order of results shown in lines 207 and 215-216, along with the data layout in Figure 1, lacks a distinct logical structure, potentially complicating interpretation for readers. I suggest rearranging the presentation to highlight essential epidemiological, magnitude, or geographic trends, which will enhance clarity and coherence.**

Response 15.1

Thank you for your comment. We opted not to make this variable binary to better distinguish between differing levels of risk. Those who own their own boots (n=210) have the ability to protect themselves against standing water at all times, it is just a matter of whether they choose to wear them/ understand the value of protection. Those without boots (n=1085) have no opportunity for the increased protection that boots offer over sandals or other shoe types. However, those who have the option to borrow boots (n=19) pose an interesting situation. It is equally possible that someone with access to borrow may not have be able to use them at key times (e.g. during heavy rains or when there is a lot of standing water the owner may want to use the boots for themselves) or that owner may not have a strong need to use the boots often, leaving the borrower open usage of the additional protection. We felt it important to leave open the potential differences within this group separate so as not to skew results in either binary grouping.

15.2

Thank you for the opportunity for us to increase clarity in the reading of this section. To aid in improving the readability of the Results, we have adjusted the subheadings to ‘Description of Study Population” (line 213), ‘Seroprevalence’ (line 232), ‘Age and Sex Stratified Seroprevalence’ (line 248), ‘Univariable analysis’ (line 258), ‘Multivariable Analysis- Model Selection’ (line 296), and ‘Multivariable Risk Factors’ (line 308). Figure 2 (formerly Figure 1) has been reformatted for clarity and is in line 242 in the Seroprevalence section. Additional information on the reformatting of this map can be found at the end of this document.

**Reviewer #3:**

**Reviewer Comment 16:**

**The manuscript presents interesting data on factors that may increase the risk of exposure to arboviruses in poor communities, such as the degree of urbanization and flood risks. However, much of the data is described in a fragmented way and presented in tables as a whole, making it difficult to understand. Some changes in the way it is presented or described would improve the fluidity of the reading and understanding.**
Response 16: Thank you for your comment. In response 15.2 we discussed edits to the structuring of this section to improve readability. While some tables were used, we also visualised other factors such as age and sex trends in seroprevalence and seroprevalence by study area in this section in other formats.

**Reviewer Comment 17:**

**(1) Lines 197-199: “Overall, study participants of all areas were relatively young, with median ages ranging from 26 in RS to 38 in MR, and an overall median age of 33 (IQR 19-48 years).” Where this data is located, which table? Please facilitate identification.**

Response 17:

Thank you for this. We have added Supplementary Table 2 which provides the median age for each community.

**Reviewer Comment 18:
18.1 Lines 203-205:”MR had a mean of 49.62m (IQR 44.0-55.4m) above sea level, AC was 54.74m (IQR 45.0-61.76m), RS was 64.65m (IQR 57.54- 73.00), but NC was far lower than other communities at 8.54m (IQR 4.83- 12.21m)”. Where this data is located, which table? Please facilitate identification.**

**18.2 Lines 205-208: “the proportion of participants in each community that reported living in a household on a hillside, with only 3.3% of participants reporting this in NC compared to 27.6%, 20.8% and 59.1% in MR, AC and RS, respectively.” Please, mention where this information is found (supplementary table 2), and in the table include a column with the percentage, to facilitate reading.**

Response 18:

18.1 Thank you for this, we have added Supplementary Figure 4 which provides a box and whisker plot of the height above sea level in meters for each community and their respective IQR.

18.2 Thank you for this, we have added Supplementary Table 4 which provides the number of individuals living on hillsides and their seroprevalence in comparison to those who do not reside on hillsides.

**Conclusions**

-Are the conclusions supported by the data presented?

-Are the limitations of analysis clearly described?

-Do the authors discuss how these data can be helpful to advance our understanding of the topic under study?

-Is public health relevance addressed?

**Reviewer Comment 19:**

**19.1 Reviewer #1: As a suggestion, the authors could organize the discussion paragraphs in the order of the domains presented: individual, household, and environmental.

19.2 The study assumes that transmission occurs primarily within household environments, as suggested by the proximity to environmental risk factors. However, in lines 372-374, the discussion argues that exposure beyond households—such as during daily travel—drives risk in men. This may lead to a potentially ambiguous interpretation.

19.3 Additionally, one of the cited studies [10], conducted in a nearby city (Feira de Santana), found a higher risk in women. Could you incorporate a discussion contrasting these findings to provide a broader perspective on possible gender-related differences in exposure?**

Response 19

19.1 Thank you for this. While individual, household, and environmental domains are how we separated the variables for analysis, we wanted to use the conclusion section to discuss the interrelation of these domains as they relate to disease transmission. However, to aid in understanding of which domains the indicators come from, we have added additional clauses in lines 357 (From our findings in the environmental domain, the higher seroprevalence in NC may also be explained by the community’s general topography and elevation relative to nearby communities), 398 (Within the household domain, the finding that houses located on hillsides had a higher risk of…), and 409 (In terms of the individual domain, we found that men were more likely to be CHIKV seropositive…).

19.2 Thank you for highlighting this. The environment close to the household is incredibly important, and our results suggest transmission takes place there (both inside and near the household). Additionally, spending additional time outside in high-risk environments such as the communities in our study areas can lead to increase exposure to mosquitos and disease transmission. We have clarified this in lines 413-416 which now reads “ In communities at high risk for CHIKV, there is risk of mosquito exposure close to home, but travelling long distances around communities in areas with high environmental risk or working outside can further increase exposure to mosquitoes away from the household and may be an explanation for greater seroprevalence in men compared to women.”

19.3 Thank you for this useful comment, the Feira de Santana paper had a majority of female participants, and in their article wrote “Our study has limitations due to the refusal to participate (22.1%) that can difficult the generalization of the results. As the refusal was mainly of men, it could have contributed to the encounter of greater seroprevalence among women.” Lines 419- 421 include this point and now read “A 2021 study of CHIKV in city near to the four study areas found a higher seroprevalence of CHIKV in women than men, however, the authors noted that having high rates of refusal in male participants could have biased this finding.”

Reviewer #2: (No Response)

**Reviewer Comment 20:**

**Reviewer #3:**

**20.1 The limitation of this study is the use of an anti-CHIKV IgG test to detect seropositivity for CHIKV, since these antibodies indicate a past infection or previous exposure to the virus, and not an acute or recent infection. Furthermore, the presence of IgG can persist for years, making it difficult to distinguish between recent and past infection.**

**20.2 In this case, the most appropriate technique would be the viral RNA test by RT-PCR (Reverse Transcription Polymerase Chain Reaction). This test can identify the genetic material of the virus during the first few days of infection, usually up to about a week after symptoms begin. IgM antibodies could also be used to detect recent infections, as they appear soon after the acute phase.**

Response 20:

20.1 Thank you for raising this point. While all study methodologies have limitations, the matter of IgG antibody persistence was addressed in lines 439-442 “As CHIKV-specific IgG antibodies can remain for over 12 months [38], exposure to infected mosquitoes could have taken place at a time when the environment, household, or individual risk factors we measured may not be fully representative of the potential exposure period.”

20.2 This is a great opportunity for further research. In the given study area, the proposed methodology would prove difficult as it would require active surveillance to be able to catch cases as soon as they occur. Seroprevalence using IgG is a commonly used technique in epidemiological cross-sectional studies to collect information on past exposures, and more information on why IgM was not used for this study can be found in Response 2.

**Editorial and Data Presentation Modifications?**

Use this section for editorial suggestions as well as relatively minor modifications of existing data that would enhance clarity. If the only modifications needed are minor and/or editorial, you may wish to recommend “Minor Revision” or “Accept”.

**Reviewer Comment 21:**

**Reviewer #1: As a minor revision that does not substantially alter the conclusions, I suggest improving consistency in Table 1. The authors list the category "Boot access" with subcategories (No, Own boots, Can borrow boots), while the other variables are presented in a binary Yes/No format. Could you clarify the rationale for using subcategories in this specific case? To ensure uniformity, I recommend standardizing all variables as dichotomous.**

Response 21: Great point, please see Response 15.1 for rationale on the division of this variable.

Reviewer #2: (No Response)

**Reviewer Comment 22:**

**Reviewer #3: Suggestions:
22.1 Clarify in the methodology or at the beginning of the results that “of the 1318 participants who provided a blood sample, 1316 had conclusive serological results and that not all answered all the questions”, to clarify the inconsistency in the total number of participants (%) in Table 1, and why the description of the result says 1,318 and the title of the table says 1,316.

22.2 When talking about the collection period (between March and October 2018), explain the importance/impact of this period in increasing exposure to CHIKV. Example: is it a rainy season in this region? Does the climate during this period favor the proliferation of the vector and consequently exposure to CHIKV?**

Response 22:

22.1 Thank you for the opportunity to increase clarity. This statement was previously listed as the first sentence of the second paragraph of the results (line 233), but I have added a variation of this information to the first paragraph as well. Line 214-216 now read “In the four communities, there were 2590 eligible individuals, of which 1318 (50.9%) consented to join the study and provided a blood sample for testing, and 1316 (99.8%) had conclusive serological results.”

22.2 This is an excellent opportunity for further research. As this is a seroprevalence study, we were not collecting information specific to temporal or climate trends.

**Summary and General Comments**

Use this section to provide overall comments, discuss strengths/weaknesses of the study, novelty, significance, general execution and scholarship. You may also include additional comments for the author, including concerns about dual publication, research ethics, or publication ethics. If requesting major revision, please articulate the new experiments that are needed.

**Reviewer Comment 23:**

**23.1 Reviewer #1: The research provides valuable insights from a multidisciplinary and cross-sectional perspective. Its strengths lie in recognizing that environmental factors have a more significant impact on risk than individual-related factors. The findings highlight the crucial role of public management in overseeing urban areas, particularly informal settlements that house marginalized communities vulnerable to CHIKV
23.2 Although these are widely recognized concepts, the authors may consider citing studies that characterize the structural deficiencies in informal settlements and how these features influence vector ecology, particularly in the sentence starting from lines 61 to 64. There are a few references used in the discussion that could be used here.
23.3 The sentence starting at lines 76-80 appears to have excessive repetition of the term 'environmental features.' I would recommend revising it to improve readability. Additionally, it would be helpful to standardize the hyphenation of the term 'fine-scale' found in lines 72 and 82.
23.4 In line 86, I suggest reviewing whether the term 'microenvironment' is the most appropriate to describe the habitat surrounding the households.**

Response 23

23.1 Thank you for your comment!

23.2 Thank you for raising this. A new reference has been added to support lines 60-64. This reference is cited as

8.	Charlesworth, S. M., Kligerman, D. C., Blackett, M., & Warwick, F. (2022). The Potential to Address Disease Vectors in Favelas in Brazil Using Sustainable Drainage Systems: Zika, Drainage and Greywater Management. International journal of environmental research and public health, 19(5), 2860. https://doi.org/10.3390/ijerph19052860

23.3 Thank you for this comment. In addressing reviewer comment 7, I addressed the wording to limit use of the phrase “environmental features”. Please see Response 7 for more information on this change. The phrase fine-scale has now been hyphenated throughout the text.

23.4 Thank you for raising this, the phrasing has been changed in 71-72 changed to from “microenvironment” to “peri-domestic environment”.

Reviewer #2: (No Response)

**Reviewer Comment 24**

**Reviewer #3: The study presents findings that reinforce the association between a higher number of breeding sites and seropositivity and environmental deficiencies such as lack of water coverage and risk of flooding. It also addresses important concerns about how environmental deficiencies and inadequate housing can increase the risk of exposure to arboviruses. In addition, it presents relevant data that can be presented to local authorities to take measures to improve sanitation and housing conditions in the region.
On the other hand, some results could be presented more clearly to facilitate reading and understanding. The study also acknowledges its limitations, which is important, but these do not completely invalidate the findings. Nevertheless, they suggest that future research could delve deeper into these issues, taking these points into account.**

Response 24: Thank you for your commentary!

In addition to the above edits to improve our article in line with the reviewers’ suggestions, we have also improved the Figure 2 (formerly Figure 1) map. The update to this map did not change its content, and the formatting changes were made to improve clarity. An additional map (Figure 1) was also added to provide additional context to the study areas and surrounding geography. Juliet Santana was added as an author for her contribution to the reformatting of these maps. Additional information on these maps and their creation is found on lines 151-158- “The maps depicting study areas and the spatial distribution of the cases were generated from the analysis of primary data produced by the authors. The data used as a basis for the creation of all the maps, including the location map of the study areas, are not protected by copyright, as they were created by the authors themselves using the free and open source software QGIS, version 3.38.3 [17].The municipal and state boundaries [22], the Orthophoto and the Digital Terrain Model, with Coordinate System: UTM, 24S time zone; Geodetic Reference System: SIRGAS 2000 [23] are also open data and were downloaded from the website of the Brazilian Institute of Geography and Statistics [22] and the City Hall of Salvador [23].”
